# Inhibition of Different Pain Pathways Attenuates Oxidative Stress in Glial Cells: A Mechanistic View on Neuroprotective Effects of Different Types of Analgesics

**DOI:** 10.22037/ijpr.2021.114476.14871

**Published:** 2021

**Authors:** Mohammad Reza Eskandari, Parivash Eftekhari, Samin Abbaszadeh, Maryam Noubarani, Bijan Shafaghi, Jalal Pourahmad

**Affiliations:** a *Zanjan Pharmaceutical Nanotechnology Research Center (ZPNRC), Zanjan University of Medical Sciences, Zanjan, Iran. *; b *Department of Pharmacology and Toxicology, School of Pharmacy, Zanjan University of Medical Sciences, Zanjan, Iran. *; c *Department of Toxicology and Pharmacology, School of Pharmacy, Shahid Beheshti University of Medical Sciences, Tehran, Iran. *; d *Department of Pharmacology, School of Medicine, Zanjan University of Medical Sciences, Zanjan, Iran.*

**Keywords:** Neuropathic pain, Mitochondria, Lysosomes, Aspirin, Celecoxib, Morphine, Etanercept

## Abstract

Neuropathic pain results from trauma or diseases affecting the central nervous system (CNS) and triggers a cascade of events in different CNS parts that eventually lead to oxidative injury. This study was aimed to investigate the protective effects of some selected analgesics in neuropathic pain-induced oxidative damage in the isolated glial cells of the rat brain. In this experiment, rats were randomly divided into 5 main groups. Rats in group 1 received no medication, whereas rats in groups 2 to 5 received ASA (aspirin), celecoxib, morphine, and etanercept daily, respectively. Each main group divides into 3 subgroups: normal, sham, and neuropathic pain model rats. The glial cells of the rat brain were isolated at different time points. Our results demonstrate that neuropathic pain induces ROS generation as the major cause of mitochondrial membrane potential collapse (%∆Ψm) and lysosomal membrane rupture, which result in oxidative damage of the glial cells. In addition, ASA and celecoxib had protective effects on the neuropathic pain-induced oxidative stress markers, including ROS production, mitochondrial membrane potential collapse, and lysosomal membrane leakiness at different time points. Furthermore, the oxidative damage markers were significantly decreased by morphine and etanercept in all investigated days. Since arachidonic acid metabolites and TNF-α are produced during neuropathic pain and inflammation, it can be concluded that the inhibition of the substances production or inhibition of the ligands binding with their receptors would help to decrease the destructive effects of neuropathic pain in the glial cells of rat brain.

## Introduction

Neuropathic pain is determined as a pain usually caused by damage or impairment of the somatosensory nervous system function, with limited therapeutic options available (1-4). Since most of the available treatments are undesirable, pain mechanisms should be investigated to provide more effective therapeutic targets. 

Glia or glial cells have been recently shown to play an essential role in neuropathic pain and as a result, are considered as a new target for the treatment (5, 6). It seems that glia activation in the spinal cord leads to pathological pain in several pain syndromes with various etiologies such as the progression of neuropathic pain, trauma, diabetic neuropathy, and spinal cord inflammation as well as peripheral nerve inflammation (6-8). A principal mechanism in pain sensation is neuroinflammation at the injury site that occurs in the acute phase after injury (9). Glial cells produce proinflammatory factors such as matrix metalloproteinases, cytokines, and reactive oxygen species (ROS) during the process of neuroinflammation (6). Several studies have proven that oxidative stress can have detrimental effects and consequences on cell function, resulting in inflammation, pain, and cell death (10, 11). Oxidative stress in biological systems is an imbalance between the overproduction of ROS (free radicals) and enzymatic and nonenzymatic antioxidant defenses systems. Besides, it is strongly believed that increased ROS formation constitutes the main risk factor for tissue injury and organ dysfunction (12).

Cyclooxygenases (COX) and prostagla-ndins play an important role in inflammatory diseases and contribute notably to the accompanying pain sensitization (13). Studies have also shown that increased levels of TNFα, as an inflammatory cytokine, may be implicated in the pathogenesis of neuropathic pain (14). Furthermore, analgesic opioids are also used to relieve neuropathic pain (15). COX2-selective, nonselective nonsteroidal anti-inflammatory drugs (NSAIDs), morphine, and etanercept are among the drugs used in relieving neuropathic pain (16-18). However, the exact cellular mechanisms involved in the prevention role of analgesics in cellular and molecular alteration induced by neuropathic pain have not yet been fully understood. Therefore, the present study aimed to evaluate the mechanisms of protective effects of some selected analgesics, including aspirin (ASA), celecoxib, morphine, and etanercept, on oxidative damage of rat brain glial cells induced by neuropathic pain.

## Experimental

Dichlorofluorescin diacetate, rhodamine 123, and acridine orange were purchased from Sigma-Aldrich Co. (Taufkirchen, Germany). All other chemicals were of the highest commercial grade available.

*Animal*

Male Sprague-Dawley rats (200-300) g were used in this study. The rats were given a standard normal food and water *ad libitum* and were housed in the ventilated plastic cages over PWI 8-16 hardwood bedding at a controlled environmental temperature of 22 ± 1 °C with 50 ± 10% relative humidity and a 12-h light/12-h dark cycle. Principles of laboratory animal care (NIH publication No. 85-23, revised 1985) were followed. All experiments were conducted according to the ethical standards and protocols approved by the Committee of Animal Experimentation of Shahid Beheshti University of Medical Sciences, Tehran, Iran. (Protocol approval number: IR.SBMU.REC.1385.221).

*Induction of neuropathic pain*

Neuropathic pain was induced in the rats according to the protocol described previously by Decosterd et al. with a few modifications (19). The Spared Nerve Injury (SNI) method included an axotomy and ligation of the tibial and usual peroneal nerves residue the sural nerve entire. Ultimately, the nerves were flattened and transparent. Sham controls involved exposure of the sciatic nerve and its branches without any lesion.

*Mechanical hyperalgesia*

After SNI, pain evaluation was performed to confirm allodynia or hyperalgesia in the rat model. With the rats on the higher grid, a pin-prick test was done by a safety pin. The lateral part of the plantar surface of the paw was briefly stimulated at a strong enough to indent but not penetrate the skin (pin-prick test). The duration of paw withdrawal was recorded, with an arbitrary minimal time of 0.5 s (for the brief normal response) and a maximal cut-off of l0 s (19). Our results confirmed the induction of hyperalgesia in the neuropathic pain rat model (Data not shown).

*Experimental protocol*

In this experiment, rats were randomly divided into 5 main groups of 72 rats each. Animals in group 1 received no medication, whereas rats in groups 2 to 5 received ASA (100 mg/kg, p.o. or orally), celecoxib (10 mg/kg, p.o.), morphine (5 mg/kg, i.p. or intraperitoneal), and etanercept (5 mg/kg, i.p.) daily, respectively (20-23). Each main group was divided into 3 subgroups as follows: 24 rats in the control subgroup as normal controls without any surgery; 24 rats in the sham surgery subgroup that the SNI surgery was performed and the rats were exposed to the trunk and branches of the sciatic nerve without ligation, and 24 rats in case or neuropathic pain model subgroup that the SNI surgery was performed along with ligated and cut oﬀ the simple peroneal and tibial nerves. The analgesics administrated daily 24 h before the experiment. In each group, the glia cells of the rat brain were isolated on the second, 6th, 10th, and 14th days after the surgery (n = 6). 

*Glial cells isolation*

The microglia were taken on the second, 6th, 10^th^, and 14th days after the surgery in each group. For this purpose, the brain was removed and embedded in PBS buffer, then hemispheres were separated, and the bean-shaped hippocampi were isolated. Hippocampus was gently minified in dishes containing PBS and then incubated in a buffer contains trypsin-EDTA for 5 min at 37 °C. Then, it was shacked gently and again incubated for 5min additionally. Then, DMEM medium was added. In order to remove neurons and oligodendrocytes, the prepared suspension was passed through the funnel (70 mesh, 25 microns) by the vacuum pump. The cell suspension was then placed in a bioreactor for 15 min. Then, cells stained with trypan blue were visualized under an optical microscope at 40 magnification. Live cells were seen in yellow and dead cells in blue. The cell viability was more than 95%. Then, 1 mL of the obtained microglia from each group was centrifuged for 1 min at 1000 rpm. The deposited was used as a cell sample to measure ROS production, mitochondrial membrane potential decline, and lysosomal membrane injury (24).

*Glial cell identification*

To confirm that a majority of isolated cells were glial cells, cells were analyzed for the expression of astrocyte marker glial fibrillary acidic protein (GFAP) through antibody staining. GFAP immunostained samples confirms that 90% of cells present are GFAP positive cells (Immunostaining data not shown).

*Determination of ROS production*

Dichlorofluorescin diacetate (DCFH-DA), a non-fluorescent compound, was used to measure the rate of ROS formation in glial cells. Dichlorofluorescin (DCFH), a non-fluorescent compound, is derived from the hydrolysis of this reagent by cellular esterase. DCFH, after reacting with ROS within the cell, produces the highly fluorescent dichlorofluorescein (DCF) that outflow the cell. For this purpose, 3 mL of cells were centrifuged for 1 min at 1000, then mixed with 3 mL of DCFH diacetate (1.6 µM). Suspension shook softly and incubated for 10 min at 37 °C. ROS levels were measured according to fluorescence severity by the fluorescence spectrometer (Excitation: 500 nm, Emission: 520 nm) and expressed as fluorescent severity per 10^6^ cells (12).

*Mitochondrial membrane potential coll-apse (%∆Ψm) measurement*

The cationic fluorogenic probe Rhodamine 123 was used to quantify mitochondrial membrane potential reduction (25). For this purpose, 0.5 mL of cell suspension was centrifuged at 1000 rpm for 1 min. The supernatant was discarded, then 2 mL of Rhodamine (1.5 µM) was added to the precipitated cells. The suspension was incubated for 10 min at 37 °C. Then, fluorescence intensity was defined at Excitation: 490 nm and Emission: 520 nm by a spectrophotometer. The capacity of mitochondria to take up the Rhodamine 123 as the fluorescence difference between the control and test cells indicates the mitochondrial membrane potential collapse and the degree of mitochondrial damage. 

*Assessment of lysosomal membrane stability*

Measurement of lysosomes membrane stability was done by acridine orange dye (26). In this method, 0.5 mL of the cell suspension was centrifuged at 1000 rpm for 1 min, and the supernatant was discarded. Then, acridine orange (5 µg/mL) was added to the cells, and the suspension was incubated for 10 min at 37 °C. Then, the fluorescence intensity from the distribution of acridine orange to the cytosol was determined at wavelength Excitation: 495 nm: and Emission: 530 nm using a spectrophotometer.

*Statistical analysis*

Levene’s test was used to check the homogeneity of variances. Data were analyzed using a one-way analysis of variance followed by Tukey’s HSD as the post hoc test. Results were presented as mean ± SD. Any differences between the groups were considered significant at *P *< 0.05.

## Results

*Effects of ASA, Celecoxib, Morphine, and Etanercept on ROS formation in neuropathic pain*

**Figure 1:**
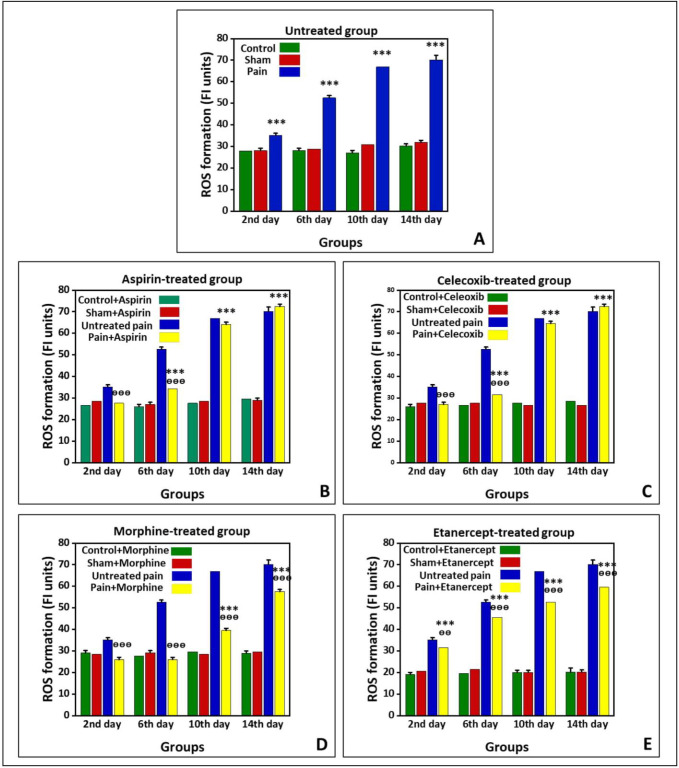
Preventing neuropathic pain-induced ROS formation by different analgesics. ROS formation is expressed as Fluorescence intensity (FI) units. (A) Untreated group, (B) ASA, (C) celecoxib, (D) morphine, and (E) etanercept on the second, 6th, 10th, and 14th days after induction of neuropathic pain. Data were presented as mean  ±  SD. ^***^P < 0.001 in comparison to the sham group; ^ᶱᶱ^*P* < 0.01, ^ᶱᶱᶱ^*P* < 0.001 in comparison to the untreated pain group

Figure 1 shows the production of ROS in the untreated group and groups treated with ASA, celecoxib, morphine, and etanercept on the second, 6th, 10th, and 14th days after induction of neuropathic pain. As shown in Figure 1A, ROS production in the neuropathic pain model rats was statically higher than the sham group in the untreated animals (*P < *0.001). The results also indicated that the ROS formation reached its highest level on the 14th day in the neuropathic pain model rats. However, there was no difference between sham and control groups on different days after neuropathic pain induction (*P > *0.05).

As shown in Figure 1B, the generation of ROS in the ASA-treated pain group did not have a considerable difference with the sham rats (*P > *0.05) on the second day after induction of neuropathic pain. However, this alteration was significant (*P < *0.001) on the 6th, 10th, and 14th days. The ROS generation reached its highest level in the case group on the 14th day. Additionally, the results confirmed that the amount of ROS produced in the ASA-treated neuropathic pain model animals was markedly lower than that of the untreated rats (*P < *0.001) on the second and 6th days. However, there was no considerable difference in ROS production between the ASA-treated pain group and the untreated group on the 10^th^ and 14th days (*P > *0.05). There was also no considerable difference between sham and control rats on different days after neuropathic pain induction (*P > *0.05).

The production of ROS in the celecoxib-treated neuropathic pain model animals did not statically differ from the sham rats (*P > *0.05) on the second day after induction of neuropathic pain (Figure 1C). On the other hand, ROS formation was markedly elevated compared to the sham rats (*P < *0.001) on the 6th, 10th, and 14th days. Also, there was no significant difference in ROS generation between sham and control groups on different days after induction of neuropathic pain (*P > *0.05). In addition, ROS production in the celecoxib-treated neuropathic pain model animals was markedly lower than in the untreated rats (*P < *0.001) on the second and 6th days. However, there was no difference in ROS production between the celecoxib-treated pain group and the untreated group (*P > *0.05) on the 10th and 14th days.

The production of ROS in the morphine-treated neuropathic pain model rats had no considerable difference with the sham animals (*P > *0.05) on the second and 6th days after induction of neuropathic pain (Figure 1D). Nevertheless, ROS formation was elevated compared to the sham rats (*P < *0.001) on the 10th and 14th days. As shown in Figure 1D, the ROS generation reached its highest level in the case group on the 14th day. There was no considerable difference between sham and control rats on different days after induction of neuropathic pain (*P > *0.05). Our results also demonstrated that the ROS production in the morphine-treated pain group is markedly lower than that in the untreated animals (*P < *0.001).

The results showed that ROS formation in the etanercept-treated neuropathic pain model rats was higher than the sham animals (*P < *0.001) at different days after the induction of neuropathic pain (Figure 1E). Similarly, ROS generation in the etanercept-treated pain group reached its highest level on the 14th day in the case group. Production of ROS was statically lower in the etanercept-treated neuropathic pain model animals than in the untreated rats on the second (*P* < 0.01), 6th, 10th, and 14th (*P* < 0.001) days. However, there was no considerable difference in ROS production among sham and control rats (*P* > 0.05).

*Effects of ASA, Celecoxib, Morphine, and Etanercept on the mitochondrial membrane potential collapse (%∆Ψm) in neuropathic pain*

**Figure 2:**
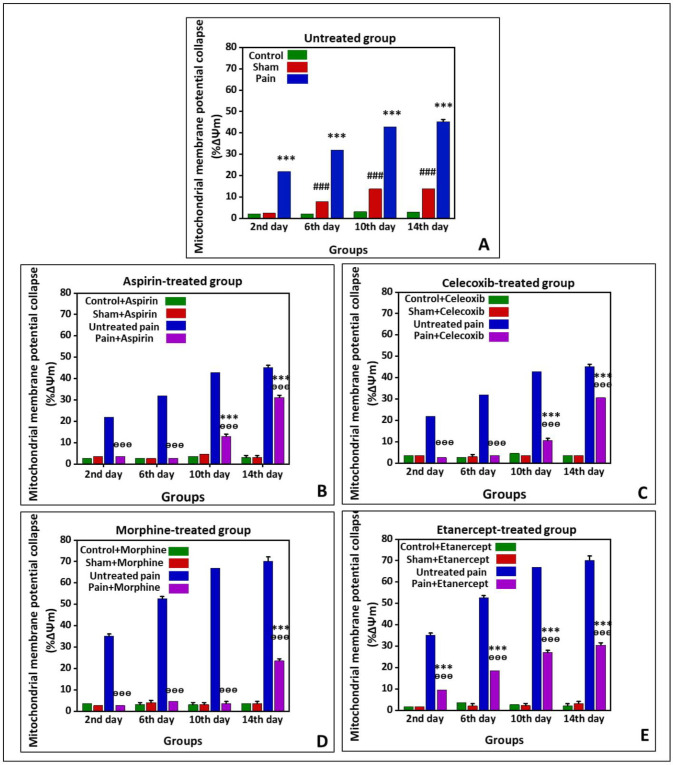
Preventing neuropathic pain-induced mitochondrial membrane potential collapse (%∆Ψm) by different analgesics. Mitochondrial membrane potential was measured as the difference in mitochondrial uptake of the Rhodamine 123 between control and treated cells and expressed as ﬂuorescence intensity unit. (A) Untreated group and groups treated with (B) ASA, (C) celecoxib, (D) morphine, and (E) etanercept on the second, 6th, 10th, and 14th days after induction of neuropathic pain. Data were presented as mean  ±  SD. ^###^*P < *0.001 in comparison to the control group; ^***^P < 0.001 in comparison to the sham group; ^ᶱᶱᶱ^*P < *0.001 in comparison to the untreated pain group

Figure 2 shows %∆Ψm in the untreated group and the groups treated with ASA, celecoxib, morphine, and etanercept on the second, 6th, 10th, and 14th days after induction of neuropathic pain. As presented in Figure 2A, the %∆Ψm was elevated in untreated pain groups compared to the sham rats (*P < *0.001) on all investigated days, which showed an upward trend. In addition, there was a considerable difference in %∆Ψm between the sham group and the control group (*P < *0.001) on the 6th, 10th, and 14th days. 

According to the results, %∆Ψm in the ASA-treated pain group was not statically different from the sham rats (*P > *0.05) on the second and 6th days after induction of neuropathic pain (Figure 2-B); however, it was significantly increased at the 10th and 14th days (*P < *0.001). There was no difference between sham and control rats on different days after induction of neuropathic pain (*P > *0.05). Our results also demonstrated that %∆Ψm was statically lower in the ASA-treated pain animals than in the untreated case rats in all investigated days (*P < *0.001). 

As can be seen in Figure 2C, %∆Ψm in the celecoxib-treated pain group did not statically differ from the sham rats (*P > *0.05) on the second and 6th days after induction of neuropathic pain; however, this rate was elevated (*P < *0.001) on the 10th and 14th days. There was no considerable difference in %∆Ψm between sham and control rats on different days (*P > *0.05). On the other hand, %∆Ψm was decreased in the celecoxib-treated neuropathic pain model rats than in the untreated case animals (*P < *0.001). 

%∆Ψm in the morphine-treated neur-opathic pain model rats was not statically different from the sham animals (*P > *0.05) on the second, 6th, and 10th days after induction of neuropathic pain (Figure 2D). But, this rate was significantly increased (*P < *0.001) on the 14th day. There was no considerable difference in %∆Ψm between sham and control group on different days after induction of neuropathic pain. However, the mitochondrial potential collapse in the morphine-treated neuropathic pain model rats was lower than that of the untreated neuropathic pain model animals (*P < *0.001).

As shown in Figure 2E, %∆Ψm was elevated in the etanercept-treated neuropathic pain model animals compared to the sham rats (*P < *0.001) on the second, 6th, 10th, and 14th days after induction of neuropathic pain. There was no considerable difference in %∆Ψm between the sham and the control group (*P > *0.05). On the other hand, the %∆Ψm was statically diminished in the etanercept-treated pain group compared to the untreated neuropathic pain model rats on different days (*P < *0.001).

*Effects of ASA, Celecoxib, Morphine, and Etanercept on lysosomal membrane stability in neuropathic pain*

**Figure 3:**
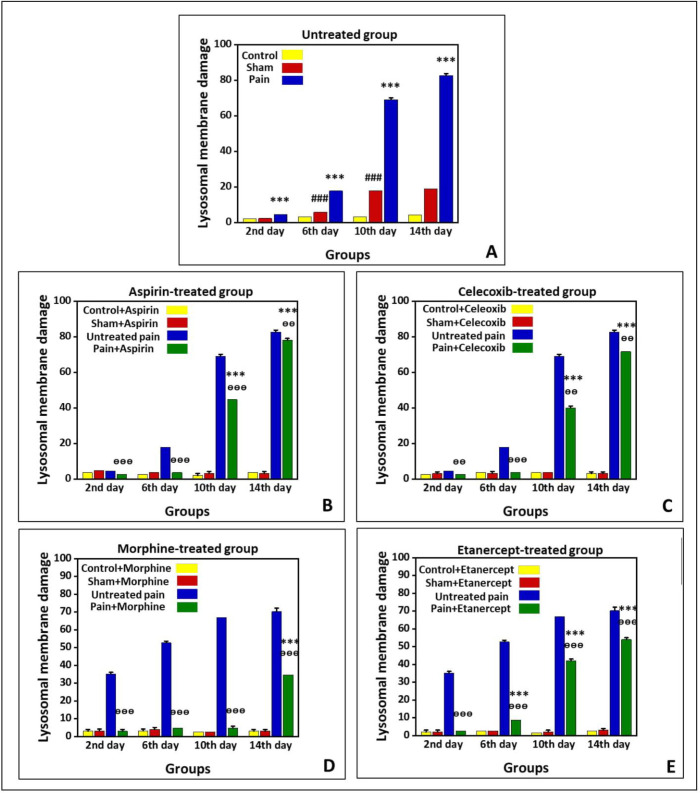
Preventing neuropathic pain-induced lysosomal membrane damage by different analgesics. Lysosomal membrane damage was measured as a difference in the redistribution of acridine orange from lysosomes into cytosol between treated cells and control cells. Our data were shown as the percentage of lysosomal membrane leakiness in all treated (test) hepatocyte groups. (A) Untreated group and groups treated with (B) ASA, (C) celecoxib, (D) morphine, and (E) etanercept on the second, 6th, 10th, and 14th days after induction of neuropathic pain. Data were presented as mean  ±  SD. ^###^*P < *0.001 in comparison with the control group; ^***^P < 0.001 in comparison to the sham group; ^ᶱᶱ^*P < *0.01 and ^ᶱᶱᶱ^*P < *0.001 in comparison to the untreated pain group

Figure 3 shows lysosomal-membrane damage in the untreated group and the groups treated with ASA, celecoxib, morphine, and etanercept on the second, 6th, 10th, and 14th days after induction of neuropathic pain. According to the results, there was significant lysosomal damage in the neuropathic pain model rats compared to the sham animals (*P < *0.001) in all investigated days, with an upward trend (Figure 3A). Besides, there was a statistical difference in the extent of lysosomal damage between the sham and control rats (*P < *0.001) on the 6th, 10th, and 14th days. 

As presented in Figure 3-B, lysosomal damage in the ASA-treated case group was not markedly different from the sham animals (*P* > 0.05) on the second and 6th days after induction of neuropathic pain. However, the extent of lysosomal damage was elevated (*P* < 0.01) on the 10th and 14th days. There was no statistical difference in the extent of lysosomal damage between the sham and control group on different days. On the other hand, the rate of lysosomal damage in the ASA-treated case animals was markedly lower than that of the untreated case rats on the second, 6th, 10th (*P < *0.001), and 14th (*P < *0.01) days. 

As shown in Figure 3C, the amount of lysosomal damage in the celecoxib-treated pain group was not markedly different from the sham animals (*P > *0.05) on the second and 6th days after induction of neuropathic pain. However, the extent of lysosomal damage was elevated (*P < *0.001) on the 10th and 14th days. There was no statistical difference in the extent of lysosomal damage between the sham and control group on different days (*P > *0.05). On the other hand, the rate of lysosomal damage in the celecoxib-treated neuropathic pain model rats was markedly lower than that of the untreated neuropathic pain model animals (*P < *0*.*001).

Our results showed that the extent of lysosomal damage in the morphine-treated pain group was not markedly different from the sham rats (*P > *0.05) on the second, 6th, and 10th days after induction of neuropathic pain. Nevertheless, it was elevated compared to the sham animals (*P < *0.001) on the 14th day. There was no statistical difference in the extent of lysosomal damage between sham and control groups on different days (*P > *0.05). On the other hand, the extent of lysosomal damage in the morphine-treated neuropathic pain model rats was lower than that of the untreated neuropathic pain model animals (*P < *0.001).

As shown in Figure 3E, lysosomal membrane damage in the etanercept-treated neuropathic pain model rats was markedly higher than in the sham animals (*P* < 0.001) on the 6th, 10th, and 14th days after induction of neuropathic pain. There was no statistical difference in the extent of lysosomal damage between sham and control groups on different days (*P > *0.05). However, the extent of lysosomal damage was lower in the etanercept-treated neuropathic pain model animals than in the untreated neuropathic pain model rats (*P < *0.001).

## Discussion

Neuropathic pain affects about 7 to 10% of people worldwide, and it can be caused by a lesion or disease of the somatosensory system, including central neurons and peripheral fibers (27). Glial cells are activated during persistent pain caused by inflammation or damage to peripheral tissues and nerves, which can subsequently release different kinds of free radicals such as ROS, nitric oxide, and proinflammatory cytokines (28). There are many therapeutic options with different drug categories for relieving neuropathic pain. The present study has investigated the efficacy of ASA (a non-selective COX inhibitor), celecoxib (a non-selective COX inhibitor), morphine (an opioid), and etanercept (a TNF-α inhibitor) against oxidative damage on rat brain glial cells in different time points after the induction of neuropathic pain. 

ROS are involved in the inflammatory response and play a key role in the destructive consequences of inflammatory reactions on tissues (10). It has been shown that in addition to inhibition of prostaglandin synthesis, the anti-inflammatory effects of NSAIDs can be due to their free radical scavenging effect. Furthermore, ROS are involved in the progress of persistent pain that results from nerve injury or inflammation (29). It has been demonstrated+ that a ROS scavenger can have an anti-hyperalgesic effect in the mouse model of SNL-induced peripheral neuropathy (29). In line with these studies, the results of the present research confirmed that neuropathic pain significantly increased the ROS production in all investigated days with an upward trend. 

ROS in pathological quantities affect two important intracellular organelles, the mitochondria and the lysosomes (30). The alteration of mitochondrial membrane potential is an essential indicator for the assessment of mitochondrial function, and it is believed that alteration in mitochondrial membrane permeability is an essential step in the development of apoptosis (31, 32). Studies have shown that damaged mitochondria produce many oxygen free radicals like superoxide (O_2_^•−^) that can diffuse into lysosomes and produce highly reactive hydroxyl radical (^•^OH). Hydroxyl radicals could destabilize the lysosomal membrane integrity and cause digestive proteases (cathepsins) released into the cytosol. These released proteases and hydroxyl radicals could open the mitochondrial permeability transition (MPT) pore and release cytochrome c that initiates downstream events, triggering caspase-3 activation and apoptosis (26). Moreover, autophagy of damaged mitochondria by lysosomes destabilizes the lysosomal membrane, and accumulating evidence demonstrated a close relationship between the autophagy-lysosome degradation pathway and neuropathic pain (33). Therefore, it can be said that the most likely source of increased ROS production in neuropathic pain is the mitochondrial damage that causes instability of the lysosomal membrane (34-36). 

One of the processes involved in cellular damage can be COX and prostaglandins. The COX pathway has specific effects on the pain process. Arachidonic acid is secreted during inflammation and is a toxic mediator and a potent stimulant for the production of ROS in brain injury. ROS are mediators of persistent pain that accompanies inflammation (37, 38). Arachidonic acid and its metabolites also stimulate microglial proliferation. For example, prostaglandin E_2_ (PGE2) can indirectly proliferate microglia (39). In this study, acetyl-salicylic acid (ASA or aspirin) as a nonselective COX inhibitor and celecoxib as a specific COX-2 inhibitor were used to control neuropathic pain. ASA and celecoxib significantly prevented ROS production on the second day after induction of neuropathic pain compared to the sham group. However, the amount of ROS production increased significantly compared to the sham group on the 6th, 10th, and 14th days. To our knowledge, neuropathic pain activates a set of damaging pathways, one of which is the COX pathway. Enhanced production of proinflammatory cytokines and nitric oxide are another important issue (28). Thus, although the COX pathway is inhibited, other pathways involved in neuropathic pain on the 6th, 10th, and 14th days have maintained the production of ROS. Therefore, the cyclooxygenase pathway appears to be one of the initiating pathways for increased ROS formation, and inhibition of COX cannot prevent the enhanced ROS production completely. As mentioned above, increased ROS formation can consequently affect mitochondrial function and lysosomes (30).

Injury of the nervous system results in neuropathic pain that is resistant to current treatments such as NSAIDs (40). However, different studies have demonstrated that cannabinoids effectively control neuropathic pain through the spinal cord and environmental mechanisms (41). In contrast, the effects of opioids in the treatment of neuropathic pain are contradictory. In the past, it was believed that neuropathic pain was resistant to opioids (42-44). However, some clinical and animal studies have confirmed that opioids can be effective in neuropathic pain control (45-47). These contradictory results can be due to different types of neuronal injury, the route of administration, potency, and selectivity of the opioid on the receptor (42). In the present study, morphine was used to assess the efficacy of opioids in neuropathic pain. The results indicated that morphine on the 14th day after induction of neuropathic pain prevented generation of ROS, mitochondrial and lysosomal membrane damages compared to the sham group. 

The results of several studies have shown that TNF-α can stimulate microglia proliferation and increase the production of ROS in different tissues (48). Studies also have demonstrated that microglial cells do not secrete TNF-α in the presence of morphine (49). However, according to our results, the efficacy of morphine was diminished on the 14th day after the induction of neuropathic pain. It was proven that neuronal injury leads to increased levels of cholecystokinin (CCK), which acts as an anti-opioid peptide. Thus, it seems that the increased CCK peptide levels may be responsible for the decrease in opioid efficacy after neuropathy (50). 

As noted earlier, glia activates and secretes cytokines, including TNF-α, following the injury of CNS (51). TNF-α, which is produced in the brain under pathological conditions, has different effects. These effects include stimulation of microglia proliferation and production of ROS (48). Since TNF-α is elevated in cellular damage following an increase in ROS production, the effect of etanercept as a TNF-α inhibitor in reducing cellular damage was investigated in this study. The ROS production and subsequent mitochondrial and lysosomal membrane damages significantly decreased in the etanercept-treated case group in all investigated days comparing to that in the untreated neuropathic pain group. However, the oxidative damage markers were increased significantly in comparison to the sham group. This is probably due to the involvement of other cytokines and proinflammatory substances that in other ways can increase the production of ROS that lead to increased lysosomal and mitochondrial damage. It should be also noted that the sham rats underwent a sham operation and only experienced surgical stress, while, in the neuropathic pain model or untreated pain model rats the SNI surgery was performed along with ligated and cutoﬀ the common peroneal and tibial nerves. Because of this surgical stress, the oxidative stress markers in the untreated pain model rats were greater than those in the sham rats. There are some other oxidative stress markers that can affect glial cells during neuropathic pain. Since, it is not possible for us to measure all of them; we believed that it was one of the limitations of the study. In addition, the lack of previous appropriate research studies on the topic is another our research limitations. 

## Conclusion

Chronic neuropathic pain induces ROS generation as the primary cause of mitoc-hondrial membrane potential decline and lysosomal membrane rupture, which result in oxidative damage in rat brain glial cells. Arachidonic acid metabolites, as well as TNF-α, are produced in inflammation. Our findings demonstrated that the inhibition of the mediators’ generation or inhibition of their binding to the receptors would help decrease oxidative damage of rat brain glial cells during neuropathic pain.
